# Altered Gray Matter Volume and Its Correlation With PTSD Severity in Chinese Earthquake Survivors

**DOI:** 10.3389/fpsyt.2018.00629

**Published:** 2018-11-30

**Authors:** Xiaoyu Zhang, Jianxin Zhang, Li Wang, Wencai Zhang

**Affiliations:** ^1^Chinese Academy of Sciences Key Laboratory of Mental Health, Institute of Psychology, Chinese Academy of Sciences, Beijing, China; ^2^Department of Psychology, University of Chinese Academy of Sciences, Beijing, China

**Keywords:** PTSD, DSM-5, gray matter volume, temporal lobe, earthquake survivors

## Abstract

**Objective:** To detect the changes of gray matter volume (GMV) and their correlation with severity of symptom in patients with post-traumatic stress disorder (PTSD) who were defined with updated DSM-5 diagnostic criteria.

**Method:** 71 participants were assigned into PTSD group (*n* = 35) or trauma-exposed control (TEC) group (*n* = 36) with the Diagnostic and Statistical Manual of Mental Disorders, Fifth Edition. Voxel-based morphometry analysis was used to detect alterations in GMV in the PTSD group.

**Results:** We found that the PTSD group had larger GMV in the left middle temporal gyrus (MTG) and in the right dorsal medial prefrontal cortex (dmPFC), and smaller GMV in the region of the right temporal pole (TP) than the TEC group. We also found that PTSD Checklist for DSM-5 (PCL-5) scores correlated positively with the left MTG and right dmPFC GMV, and negatively with left TP GMV. These correlations were consistent with the findings of the between-group comparisons.

**Conclusions:** GMV alterations in the MTG, dmPFC, and TP are detected in the group comparisons and correlated with symptom severity when classifying PTSD individuals according to DSM-5 diagnostic criteria within an earthquake-exposed population.

## Introduction

More than 70% of the people worldwide experience a traumatic event at some time in their lives, and 31% experience four or more events (1). The prevalence of post-traumatic stress disorder (PTSD) in the first year of the occurrence of natural disasters has been documented to range from ~5 to 60% (2). Due to its epidemicity and damage to health, the neural mechanisms of PTSD warrant attention.

Numerous neuroimaging studies have been conducted to identify anatomical structure of the brains of individuals classified into study groups (PTSD vs. trauma exposed control) based on the Diagnostic and Statistical Manual of Mental Disorders, Fourth Edition (DSM-IV-TR). Several meta-analysis studies found abnormal anatomy of gray matter in regions including the medial prefrontal cortex (mPFC) or anterior cingulate cortex (ACC), hippocampus, amygdala, middle temporal gyrus (MTG), temporal pole (TP), insula, and occipital cortex (3–5); the medial PFC has been implicated most frequently in previous studies. It can be divided into a more ventral portion (ventromedial prefrontal cortex, vmPFC) with a regulatory role in emotional processing and a more dorsal portion (dorsomedial prefrontal cortex, dmPFC) involved in the appraisal and expression of negative emotion (6, 7). The vmPFC is considered central to the pathophysiology of mood and anxiety disorders (8). A meta-analysis of 319 subjects (3) and studies of maltreated youths (9) and combat veterans (10) consistently found a reduction in gray matter volume (GMV) in the vmPFC in PTSD groups. Conversely, a few studies did not find reduced GMV in the PFC of maltreated children with PTSD (11, 12). In contrast, previous studies have less often reported changes in the gray matter of the dmPFC in PTSD. The role of the dmPFC can be understood by examining the other relevant literature. For example, people with PTSD show greater dmPFC activation in response to menacing scenes (13) or negative words (14), and the GMV of the dmPFC is positively correlated with the use of expressive suppression strategy (15). These evidences suggest that the anatomical and functional abnormalities of subdivisions of the mPFC in PTSD are complex and require further clarification.

Anatomical abnormalities in other brain areas are frequently reported in studies with PTSD patients. First, the hippocampus has been shown to be critical for the formation of new memories (16), and PTSD patients have deficits in declarative memory (17), which may be associated with the observed volume reduction in the hippocampus. Various cross-sectional meta-analyses have consistently identified reduced hippocampal volumes in patients with PTSD (3–5), although some other studies failed to find a smaller hippocampus in patients with PTSD (18–20). Second, the amygdala is involved in the responses to stressful experiences. Previous studies found inconsistent evidence of gray matter abnormality of the amygdala, such as greater, unchanged, and smaller gray matter volume (21–23). Third, the investigators also observed gray matter abnormalities in the MTG and TP; the MTG may contribute to the altered recall of personal memory in PTSD (24). The TP is highly interconnected with both the amygdala and orbital frontal cortex, and is thus implicated in emotion processing (25). A meta-analysis confirmed that patients with PTSD have a lower TP/MTG GMV than the trauma-exposed controls (TEC) (3). However, two other studies have shown that patients with PTSD exhibit a significantly greater right superior temporal gyrus GMV (11, 26). Therefore, it is still unclear as to how the GMV of different parts of the temporal lobe changes following the development of PTSD; additional data is required to clarify the role of the temporal lobe.

In the present study, we focus on identifying the altered GMV of brain regions when classifying PTSD individuals according to DSM-5 diagnostic criteria within an earthquake-exposed population. The individuals who were exposed to the earthquake, and then experienced symptoms of a post-traumatic syndrome and regional brain anatomy was altered. However, whether or not we detect these anatomical abnormalities scientifically rest largely in how we classify the participants included in the study into PTSD vs. no PTSD. In other words, it is important to detect the altered regional GMV with updated DSM-5 criterion, which may be different from the findings with DSM-IV criterion. PTSD has been newly defined as a trauma- and stressor-related disorder. It was found that 39 (19.1%) of the 204 respondents who satisfied the DSM-5 criteria for PTSD did not meet the criteria for a PTSD diagnosis based on DSM-IV-TR (27). Recent field studies have shown only a 55% overlap between individuals identified as having PTSD according to the DSM-IV criteria and those meeting the DSM-5 criteria (28). To date, few studies have examined how abnormalities in gray matter correlate with the DSM-5 criteria within an earthquake PTSD sample population and if these abnormalities could replicate the findings based on DSM-IV-TR criteria. An earthquake sample population is superior in terms of minimal variability in regard to the time of trauma exposure and low variability in the nature of the traumatic event, and therefore is ideal for the investigation of neural changes of PTSD based on DSM-5 criterion. Moreover, a voxel-based morphometry (VBM) method enables the assessment of anatomical differences in the whole brain and avoids operational bias toward brain structures (29), and has been used in many structural magnetic resonance imaging (MRI) studies of various neuropsychiatric disorders (30–32). Thus, the present study used the DSM-5 criteria for PTSD, a sample population of the survivors of the 2008 Wenchuan earthquake, and a VBM method to detect the abnormalities in gray matter.

Compared with previous studies, DSM-5 criteria and a larger sized Chinese Wenchuan earthquake sample population were used in the current study. Here, we intended to answer two questions: (1) what are the differences in GMV between the PTSD group and TEC group without PTSD? (2) How do altered GMV in brain areas relate to symptom severity in PTSD?

## Methods

### Participants

A magnitude 8.0 Wenchuan Earthquake occurred on May 12, 2008 in Sichuan Province, China, and killed more than 60,000 people. All participants were recruited from a single rebuilt community in Mianzhu City, Sichuan province, China, 58 km away from the epicenter of the main earthquake, and which was destroyed in the earthquake. No organized systematic intervention was available to this setup from 2008 to the time of the study. We collected our data in November 2013, approximately five and a half years after the earthquake. Seventy-one participants (mean age, 49.52 ± 6.77 years; range, 38–62 years) were included. All of them received psychological intervention by the ways of the lectures and supportive counseling, but didn't receive any organized systematic intervention from 2008 to the time of the study. The inclusion criteria were as follows: (1) household was used as the basic sample unit, and the household member whose birthday was closest to the date of investigation was first selected for participation, and if the individual was unavailable, the household member whose birthday was the next closest was selected; (2) 38–62 years, and experienced the disaster personally; (3) right-handed. The exclusion criteria were as follows: individuals with (1) mental retardation and any major psychosis (e.g., schizophrenia and organic mental disorders); (2) drug or alcohol abuse; (3) history of head trauma or surgery;(4) a metallic embedded object in body; (5) claustrophobia; (6) exposure to other trauma events from 2008 to the time of the study. The study procedures were approved by the Institute of Psychology, Chinese Academy of Sciences. All subjects provided written informed consent to participate in the experiment.

All participants were assessed in accordance with the PTSD Checklist for DSM-5 (PCL-5) (33), a trauma exposure questionnaire (34), the Center for Epidemiologic Studies Depression Scale (CES-D) (35). PCL-5 was used to assess PTSD through symptom clusters. It is a 20-item self-report measure, and is adapted from the original PCL to map directly onto PTSD's symptom criteria of the DSM-5. The Chinese version of the PCL-5 was adapted by a two-stage process of translation and back translation based on the Chinese version of the original PCL, and widely used in trauma-related research and practice (35). In this study, participants were instructed to complete the PCL-5 referring to the 2008 Wenchuan Earthquake and rate each item on a 5-Likert scale to indicate the severity of a particular symptom during the past month from 0 (not at all) to 4 (extremely). The items with symptom severity >2 points are used for diagnosis. If the participants meet at least 1 symptom criteria within intrusion symptoms, at least 1 symptom criteria within avoidance symptoms, at least 2 within negative alterations in mood and cognitions symptoms, and at least 2 within hyperarousal symptoms, they will be diagnosed as PTSD. Thirty-five participants who met the criteria for PTSD were assigned to the trauma-exposed PTSD group (PTSD group), and the remaining 36 were assigned to the trauma-exposed control group (TEC group). The self-reported score of the trauma exposure questionnaire was used to quantify the extent of trauma exposure after the earthquake. Participants were asked to provide yes (1) or no (0) responses to questions regarding post-earthquake events reflecting different levels of trauma exposure. The trauma scores range from 0 to 11 (34). The CES-D is used to assess depression symptoms. It is a self-report scale of 20 items, reflecting depressed mood, feelings of guilt or worthlessness, perceptions of helplessness or hopelessness, and psychomotor–somatic symptoms. The CES-D has been validated and widely used in Chinese populations (36).

### Data acquisition

All imaging data were collected using a 1.5 T Philips Achieva Scanner. After participants were positioned in the scanner, a T1-weighted low-resolution structural image was aligned approximately parallel to the anterior callosum-posterior callosum line. High-resolution T1-weighted anatomical images were then acquired using a three-dimensional gradient-echo sequence (repetition time = 8.5 ms, echo time = 3.74 ms, flip angle = 8°, field of view = 220 × 220 mm^2^, matrix = 220 × 200 pixels, slices = 180, slice gap = 0, slice orientation = sagittal, voxel size = 1 × 1 × 1 mm^3^). The total acquisition time was 5 min 23 s. Throughout the scanning procedure, participants were instructed to close their eyes, remain awake, and maintain immobility, especially for the head. Foam cushions and earplug were used to fix the head and block machine noise for minimizing the head motion.

### VBM-DARTEL preprocessing

Statistical Parametric Mapping 8 software (SPM8; Wellcome Department of Imaging Neuroscience, London, UK) was used to conduct the VBM analysis of the 3D T1-weighted MRI data using Matlab 2010a (MathWorks, Natick, MA, USA). First, the images for each participant were inspected for artifacts and subsequently transformed to three-dimensional Montreal Neurological Institute (MNI) space. Next, the images were segmented into gray matter, white matter, and cerebrospinal fluid (CSF). The DARTEL toolbox was used to improve inter-subject co-registration of structural MR images. For each participant, a flow field storing the deformation information for warping the participant's scans onto the template was created. These were then used to spatially normalize gray matter images to MNI space employing affine spatial normalization as implemented in the normalization algorithm included in the DARTEL toolbox. Additionally, Jacobian-scaled (“modulated”) warped tissue class images were created to conserve the total volume within the voxels. Finally, a DARTEL template was constructed with each voxel resampled to 1.5 × 1.5 × 1.5 mm and smoothed with an 8-mm full-width half-maximum (FWHM) isotropic Gaussian kernel.

### Data analysis

VBM was performed using the DARTEL algorithm in SPM8 to quantify GMV. First, the differences in GMV between the PTSD and TEC groups were assessed using a two-sample *t*-test with SPM8, with total intracranial volume (TIV), age, level of education, depression score, and trauma score as covariates. The results were reported at a threshold of *p* < 0.01 (voxel-level family-wise error [FWE] correction, continuous voxels >100). Subsequently, to detect the relationship between GMV and PTSD symptom severity, Pearson's correlation was calculated with the Resting-State fMRI Data Analysis Toolkit (REST V1.8 software, by SONG Xiao-Wei et al., http://www.restfmri.net) using TIV, age, level of education, depression score, and trauma score as covariates. The results were reported with a threshold of *p* < 0.05 (voxel-level False Discovery Rate [FDR] correction, continuous voxels >100). Other statistics analyses were calculated using IBM SPSS Statistics 20.0 for Macintosh (IBM Corp., Armonk, NY, USA).

## Results

### Subject characteristics

Demographic and clinical data are presented in Table 1. There were no significant differences between the PTSD and TEC groups in terms of age, gender, level of education, trauma scores, or total intracranial volume, but there were significant differences in the depression and PCL-5 scores.

**Table 1:** Demographic and clinical information of study subjects.

**Characteristic**	**TEC group**	**PTSD group**	**T/X^2^**
Handedness (right/total)	36/36	35/35	/
Age (mean years/SD)	48.22/6.75	50.86/6.62	−1.66
Gender (male/female)	19/17	14/21	0.35
Education (mean level/SD)	2.6/1.14	2.26/0.92	1.38
Depression (mean scores/SD)	36.90/8.92	45.48/9.49	−3.92^***^
Trauma (mean scores/SD)	5.36/1.26	4.74/1.65	1.77
PCL-5 (mean scores/SD)	18.06/8.76	43.72/10.58	−11.14^***^
Adaptive strategy (mean scores/SD)	29.42/6.25	31.69/6.61	−2.21
Maladaptive strategy (mean scores/SD)	17.90/5.16	25.07/5.59	−23.36^***^
Total intracranial volume (# voxel amount/SD)	1,855,272.6/184,220	1,824,432.9/169,506	0.73

*** p < 0.001. SD, standard deviation.

# voxel size = 1 × 1 × 1 mm^3^.

*Educational level: one corresponds to he (or she) finished primary school; two junior school; three senior school; four polytechnic school; five college; and six post-graduate*.

### GMV differences between PTSD and TEC groups

The PTSD group had a larger GMV in the bilateral middle temporal gyri, right dorsomedial prefrontal cortex, and right posterior cerebellar lobe, and a smaller GMV in the right temporal pole than the TEC group. These results are presented in Table 2.

**Table 2:** Comparison of GMV between PTSD and TEC groups.

**Brain region**	**Cluster size**	**MNI**			***Z* score**
		***X***	***Y***	***Z***	
**PTSD** > **TEC**					
Left Temporal_Mid _(aal)_/Superior temporal gyrus	500	−52	−48	0	7.74
Right cerebellum posterior lobe	295	5	−70	−51	7.70
Right Temporal_Mid _(aal)_/Superior temporal gyrus	370	54	−42	−2	7.48
Left Frontal_Mid _(aal)_/Frontal_Inf_Tri _(aal)_	176	−39	38	21	6.41
^*^Right Frontal_Mid _(aal)_/Dorsomedial frontal gyrus	201	33	47	15	4.91
**PTSD**<**TEC**					
RightTemporal_Pole Sup _(aal)_/Temporal_Pole_Mid _(aal)_	203	48	24	−29	-5.10

(aal) denotes automated anatomical labeling of the macroscopic brain structure. The results labeled (aal) were reported at a minimum cluster size of 50 continuous voxels with a threshold of p < 0.01 (voxel-level family-wise error [FWE] correction) in each aal template.

* *means this result was reported at a threshold of p < 0.05, FWE correction*.

### Correlation between global GMV and PCL-5

**Figure 1:**
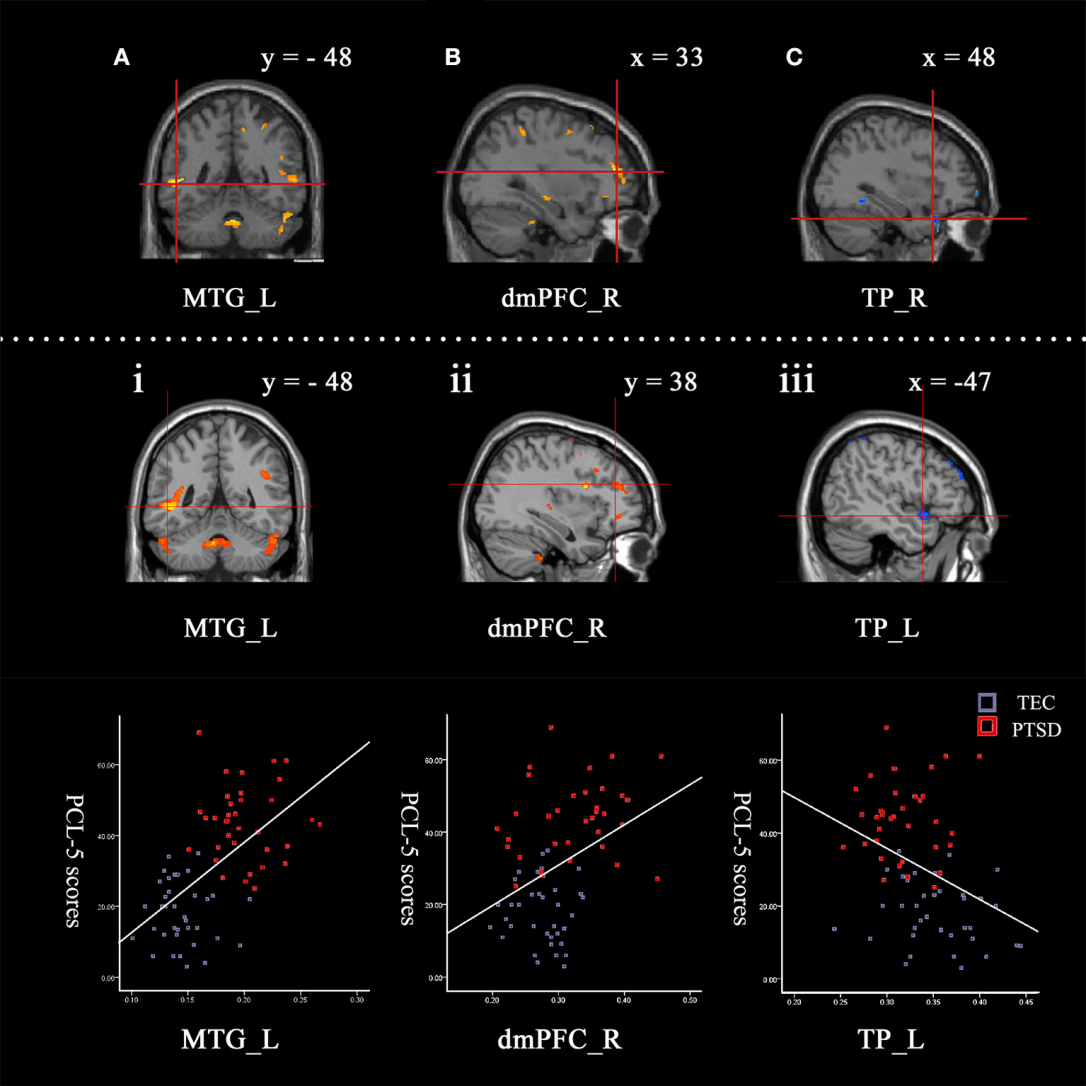
Contrasts between regions in the between-group comparisons (above the dotted line) and regions correlated with PCL-5 scores among all participants (under the dotted line). Between-group comparisons: **(A)** The left middle temporal gyrus (−52, −48, 0) and **(B)** right dorsomedial prefrontal cortex (33, 47, 15) had a larger GMV in the PTSD group (PTSD > TEC). **(C)** The right temporal pole (48, 24, −29) had a smaller GMV in the PTSD group (PTSD < TEC). These are very close to the findings of the correlative analyses between GMV and PCL-5 scores with all participants. The GMVs of the following regions: **(i)** The left middle temporal gyrus (−47, −48, −2; *r* = 0.69) and **(ii)** right dorsomedial prefrontal cortex (33, 38, 21; *r* = 0.53) were positively correlated with the PCL-5 score. The GMV of **(iii)** the left temporal pole (−47, 9, −14; *r* = −0.55) was negatively correlated with the PCL-5 score. The signal values are extracted to depict the graphs of the correlation in these regions. The cross indicates the peak-values of clusters. L refers to left cerebrum, R refers to right cerebrum.

The correlations between global GMV and total score of PCL-5 were computed. Among all participants, PCL-5 total scores were positively correlated with the GMV in the left middle temporal gyrus, left middle occipital gyrus, and right dorsomedial prefrontal cortex, and negatively correlated with the GMV of the left temporal pole and left anterior cingulate. These results were consistent with the findings of the between-group comparisons. The results are shown in Table 3 and Figure 1.

**Table 3:** Correlation between global GMV and PCL-5 scores.

**Brain region**	**Cluster size**	**MNI**			***r***
		***X***	***Y***	***Z***	
**POSITIVE CORRELATION**					
Left Temporal_Mid _(aal)_/Superior Temporal Gyrus/Frontal_Mid _(aal)_	2,545	−47	−48	−2	0.69
Left Occipital_Mid _(aal)_/Middle temporal gyrus	306	−30	−75	3	0.59
Right Frontal_Mid _(aal)_/Dorsomedial prefrontal cortex	230	33	38	21	0.53
**NEGATIVE CORRELATION**					
Left Temporal_Pole_Sup _(aal)_/Temporal_Sup _(aal)_	127	−47	9	−14	−0.55
Left Cingulum_Mid _(aal)_/Anterior cingulate	275	−12	2	33	−0.58

*The results labeled (aal) were reported at a minimum cluster size of 100 continuous voxels with a threshold of p < 0.01 (voxel-level FDR correction) in each aal template*.

## Discussion

The present study used DSM-5 criteria to define PTSD symptom severity, and then compared the regional GMV between PTSD and TEC groups and examined the relationship between GMV and PTSD symptom severity. We observed: (1) increased GMV in the dmPFC and MTG, and reduced GMV in the TP were found in the PTSD than the TEC group; (2) the GMV changes in these regions correlated with PTSD symptom severity.

We found increased GMV in the dmPFC, and its positive correlation with PTSD symptom severity. Anatomical studies have found that emotion dysregulation is related to greater dACC surface area in traumatized civilians (37). Increased thickness of dACC has been observed in MDD patients compared with that in controls (38, 39). In healthy adults, greater dACC thickness is positively related to skin conductance response during fear conditioning (40). Greater GMV of the dmPFC is positively correlated with the use of maladaptive expressive expression strategies (15). In contrast, only one study with DSM-IV found decreased GMV in the left dmPFC in PTSD (41). Additionally, functional studies also found that increased dmPFC activation is associated with PTSD; dmPFC activation is positively correlated with PTSD symptom severity and depression symptoms (37, 42, 43). Greater dmPFC activation in fMRI tasks involving menacing scenes (13) and negative words (14) is observed. Activation of dmPFC is also highly correlated with temperament dimension harm avoidance (44) and may also be associated with avoidance success modulated (45). There is growing evidence that supports the idea that volumetric changes can occur based on usage (46–48). Therefore, it is possible that greater GMV in the dmPFC is a reflection of greater use of this region and greater emotion dysregulation, such as excessive negative and depressive emotion expression, maladaptive avoidance, and expression suppression. Etkin and his colleagues reviewed the role of the mPFC, suggesting that the dorsal anterior cingulate cortex and mPFC seem to function generally in the expression of fear or anxiety (7). This view is well-consistent with our findings. In comparison to DMS-IV, the newly updated DSM-5 highlighted PTSD-related negative cognition, self-denigration, and negative world views and refined the description of avoidance and considered these as two separate diagnostic clusters (49). This may be an important reason that previous studies could not find increased GMV of the dmPFC when classifying PTSD individuals according to DSM-IV diagnostic criteria.

However, we did not find decreased GMV in the vmPFC. The neurocircuitry model of PTSD proposes that pathologically elevated levels of negative affect in mood and anxiety disorders result from deficient vmPFC-mediated inhibition of amygdala activity in PTSD (50, 51). Several meta-analyses of VBM in patients with PTSD found that patients tended to have a smaller vmPFC (3–5, 52), strongly supporting the neurocircuitry model. However, some studies found differing results, such as increased GMV of the vmPFC in pediatric PTSD (12), hyperactivity during task engagement and in the resting-state (53–56), and reduced likelihood of developing PTSD with vmPFC damage (57). The first reason for the inconsistent findings may partly be the functional heterogeneity of the vmPFC (8, 43), the perigenual vmPFC could be involved in the neural modulation of emotional responses to adverse stimuli, and posterior (subgenual) vmPFC activity is positively associated with negative mood, distress of social exclusion, and anticipatory anxiety. The second reason may be the use of the DSM-5 criterion in this study, rather than the DSM-IV criterion used in most of the previous VBM studies. In addition, the influence of the nature of the trauma, duration of PTSD, and comorbidities may contribute to these inconsistences.

We observed increased MTG/STG GMV in PTSD and its positive correlation with symptom severity. Previous studies have suggested that these regions play key roles in autobiographical memory (24), and abnormalities of autobiographical memory have been implicated as a cognitive risk factor for PTSD (58, 59). In the anatomical studies, the increased GMV of the MTG/STG was found in maltreated children with PTSD (11, 26), in women with borderline personality disorder with co-occurring PTSD (60), and in the trauma-exposed participants (61). MTG/STG hyperactivity was also reported when performing an encoding and retrieval memory task in PTSD (62). Therefore, the increased GMV of the MTG may be associated with the symptom of re-experiencing spontaneous memories of the traumatic event, recurrent dreams related to the trauma, flashbacks, or other intense or prolonged psychological distress. This speculation is consistent with the opinion that abnormal memory symptoms in PTSD appear to be related to altered activity in the temporal areas during both the encoding and retrieval phase of memory processing (63, 64). However, a meta-analysis based mainly on the DSM-IV criterion found that PTSD patients show a lesser GMV of MTG than trauma-exposed controls (3); Decreased GMV of MTG was also found in anxiety disorders without major depression disorders (65–68). In the updated DSM-5, PTSD belongs to a new category, called “Trauma and Stressor-Related Disorders” and was separated from anxiety disorders. This change is reflected as increased depression-related symptoms in negative alteration in mood and cognitions and therefore reduced percentage of anxiety-related symptoms. Moreover, the PTSD sample population of this study exhibited significant co-occurring depression symptom. This change in DSM-5 may contribute much to the inconsistent findings with DSM-IV.

We found larger GMV in the cerebellum in PTSD group. Previous studies only considered the cerebellum as a classical subcortical center for motor control (69), while more recent research suggests a similar role for the cerebellum in cognitive and emotional processes through its connection with limbic structures and the hypothalamic-pituitary-adrenal axis (70). Anatomical studies revealed that, via the thalamus, the cerebellum interacts with multiple areas of the prefrontal cortex, subcortex, and limbic lobe. For example, participants with PTSD with a history of sexual assault showed increased gray matter density in the cerebellum (71). Cerebellar activation was reported in mood disorders (72, 73), and heightened activation was induced in the cerebellum, cingulate, and prefrontal cortex when performing a Stroop color-word interference task (74). These findings suggest that the cerebellum may be involved in functional compensation for the pathological changes in the neurocircuitry of patients with PTSD.

In contrast, a smaller GMV of the TP was found and negatively correlated with symptom severity of PTSD. A meta-analysis of gray matter changes in PTSD based mainly on DSM-IV criterion also reported reduced GMV in the TP (3). It indicated that this study, based on the DSM-5 criterion, replicated previous findings with the DSM-IV criterion. This reduction was suggested to be associated with a deficit in theory-of-mind and mentalizing processes (3). The TP has long been considered a part of the paralimbic region (25) and showed reduced GMV and reduced activity in the emotion cognition tasks in patients with bipolar disorder (75). It was also suggested to be involved in socioemotional processing in Klüver-Bucy syndrome, which is characterized by behaviors, such as tameness, blunted affect, diminished fear, and social withdrawal (76). Therefore, the TP may be involved in abnormal emotion processing in PTSD.

In conclusion, the present study revealed that when classifying PTSD individuals according to DSM-5 diagnostic criteria within an earthquake-exposed population, GMV is increased in the bilateral MTG/STG and right dmPFC and decreased in the right TP in the PTSD group when compared with the TEC group, and the GMV changes in these regions correlated with PTSD symptom severity. These finding provided evidences for the impact of earthquake trauma on the brain anatomy according to DSM-5 diagnostic criteria.

However, there are some limitations in the present study. First, we have attempted to map the morphological substrates of PTSD pathology according to phenomenologically defined DSM-5, which is in discordance with a system level proposed by NIMH's Research Domain Criteria (RDoC). For future studies, the RDoC framework is more suitable to understand the risk mechanisms of PTSD. Second, previous studies based on the DSM-IV-TR criterion found decreased gray matter in the regions including mPFC/ACC, hippocampus, MTG, insula, and occipital cortex, which are inconsistent with our findings. As we did not collect data according to DSM-IV criteria, we could not compare the differences between the two criteria and could not definitely answer the question of whether the epidemiological divergence in PTSD between DSM-5 and DSM-IV maps to brain-morphological differences. Third, the present study used a cross-sectional research method, hence the observed anatomical abnormalities may be explained as an antecedent risk or vulnerability factor for the exposure to a traumatic event that could then cause PTSD, or explained as a product of the course of PTSD and hence longitudinal studies are preferred for future studies.

## Author contributions

All authors have significantly contributed to the research described in this article. LW, WZ, JZ, and XZ developed/planned the experiment. XZ, LW, and WZ performed the experiments. XZ and WZ analyzed the data and wrote the paper.

### Conflict of interest statement

The authors declare that the research was conducted in the absence of any commercial or financial relationships that could be construed as a potential conflict of interest.
